# Preventing tuberculosis in healthcare workers of the radiology department: a Malaysian perspective

**DOI:** 10.2349/biij.2.1.e3

**Published:** 2006-01-01

**Authors:** LH Tan, A Kamarulzaman

**Affiliations:** Infectious Diseases Unit, Department of Medicine, University of Malaya Medical Centre, Kuala Lumpur, Malaysia

**Keywords:** Tuberculosis, health care workers, developing countries

## Abstract

Tuberculosis (TB) is a well recognised occupational hazard for healthcare workers (HCWs). Concerns on the safety of healthcare settings in Malaysia was raised following a report of 25 HCWs working in 11 general hospitals in Malaysia who were infected with TB in 2004 being publicised in the media recently. As the disease burden in general is high in Malaysia, due attention should be given to this disease in our healthcare facilities including the radiology department, an often neglected area in TB infection control programmes. This article focuses on the key control measures that can be implemented in radiology departments in a developing country with limited resources.

## INTRODUCTION

Tuberculosis (TB) is a well recognised occupational hazard for healthcare workers (HCWs) [1]. This occupational hazard has received renewed concern following numerous outbreaks of both drug-susceptible TB and multidrug-resistant TB at hospitals in the United States (US) and Europe after the onset of the acquired immunodeficiency syndrome (AIDS) epidemic [2,3]. Many preventive guidelines have been introduced in the industrialised world since then. Developing countries are not spared from this risk and are more in need of an effective infection control programme because of their much higher TB and HIV burden [4]. Recently, a report of 25 HCWs working in 11 general hospitals in Malaysia who were infected with TB in 2004 received a lot of media attention (New Straits Times July 27, 2005) and raised concerns on the safety of healthcare setting in Malaysia. In University of Malaya Medical Centre (UMMC), a teaching institution and a tertiary referral centre, 8 HCWs were reported to have active TB in 2004 (UMMC Health Care Workers TB Surveillance, unpublished). Certainly due attention should be given to this disease in healthcare facilities including the radiology department, an often neglected clinical area in TB infection control programmes. Since an active surveillance of HCWs infected with TB at UMMC was instituted thus far no radiology department staffs has been reported to be infected. Nevertheless, the radiology department staffs are at potential risk of infection and need to take the necessary precautions to minimise this risk.

## RISK ASSESSMENT

The risk of TB infection and disease among HCWs in developing countries, including Malaysia, has not been well defined. A recent study performed at the UMMC suggests that the HCWs have an increased risk of acquiring TB despite having had Bacille Calmette-Guerin (BCG) vaccination [5]. Approximately half (50.1%) of the HCWs in the survey were found to have tuberculin skin test (TST) reaction of 10 mm or more, while 26.2% had TST reaction of 15 mm or more. It was concluded that this occupational risk was associated with the level of occupational TB exposure.

Radiology area-specific risk assessment has not been well studied. However, the radiology department receives a variety of patients ranging from pediatric to geriatric age groups; and from non-immunocompromised to immunocompromised states for various diagnostic and therapeutic procedures, some with diagnosed or undiagnosed TB. Therefore, the HCWs working in this ambulatory care setting are also at risk for TB transmission.

## FUNDAMENTAL PRINCIPLES IN TB INFECTION CONTROL PROGRAMME

Numerous guidelines for preventing nosocomial TB have been formulated in developed countries. One of the most elaborate and authoritative guidelines were produced by the Centers for Disease Control and Prevention (CDC) [6]. However, implementing such guidelines can be expensive and is a heavy burden for many hospitals, and probably beyond the capacity of many developing countries, including Malaysia [7]. But together with the guideline formulated by the World Health Organization (WHO), it can serve as an important source of reference.

There are three levels of control measures that need to be integrated for proper implementation of any local TB prevention programmes (ordered according to their importance and priority):

1. Administrative controls
2. Engineering controls
3. Personal respiratory protection for HCWs

### Administrative control

Administrative control measures are primarily intended to reduce the risk of HCWs as well as patients' exposure to *Mycobacterium tuberculosis*. With the findings from the survey [5], and taking into account the local capabilities and priorities, a TB infection control policy for the whole hospital was formulated integrating the above mentioned levels of control measures. The policy was approved by the hospital authority for implementation in April 2002. The Hospital Infection Control Unit was assigned the responsibility and authority to implement and enforce this policy. This article focuses on the control measures pertaining to the radiology department.

### Patients

Priority should be given to the rapid identification, isolation, diagnostic evaluation, and treatment of patients with suspected TB because the undiagnosed case of TB has long been considered the greatest potential mechanism for dissemination of the disease [3]. A triage system should be set up to identify patients with infectious TB (both suspicious and confirmed cases). All requests from the clinicians for diagnostic and therapeutic radiological procedures should indicate clearly whether the patient whom the clinician is attending to is infectious or otherwise. Radiology counter staffs should also be trained to ask questions that will facilitate the identification of such patients.

Appropriate placement of patients with infectious TB in a respiratory isolation room has reduced the risk of infection and disease to HCWs. Therefore, an isolation room or a separate room to place patients with infectious TB while waiting for radiological diagnostic or therapeutic procedures or post-procedure should be created to reduce the risk of exposure of non-infected persons. The patient should be explained and instructed to wear a surgical mask at all times while being wheeled out from the isolation room in the ward to the radiology department. The patient should be given tissues and advised to use them should he/she cough or sneeze.

### HCWs

Surveillance of HCWs with TB should be in place as stated in the policy. All HCWs diagnosed with TB should be reported to the Hospital Infection Control Unit for continuous monitoring on the situation of each unit/area.

HCWs should implement respiratory protection during radiological procedures that may induce cough or generate aerosols (e.g., irrigation of tuberculous abscesses, etc.) from patients with infectious TB as these procedures can increase the likelihood of droplet nuclei being expelled into air.

BCG vaccination has long been introduced as part of the routine vaccination in Malaysia. Most of the HCWs would have been vaccinated by the time they are employed. If they are not, they should be encouraged to do so although the efficacy of the vaccine is known to be variable, ranging from zero to 80% in controlled trials. A recent meta-analysis showed that BCG vaccination reduced the risk of developing active disease by 50% and may be cost effective in developing countries with high prevalence of TB [8].

A pre-employment two-step tuberculin skin testing should be performed to identify HCWs who have negative TST reaction. HCWs with negative TST should be advised to take extra precautions and, if possible, not to care for patients with infectious TB. Similarly, immunocompromised HCWs should be advised not to take care of infectious patients.

Periodic TST screening to monitor the skin test conversion rate has been advocated in the CDC guidelines and elsewhere, particularly in countries with low prevalence of TB and BCG vaccination. This practice has not been implemented in the local setting of high-prevalence BCG vaccination. The vaccination has been known to cause false positive cross reactions and booster phenomenon, while complicating the interpretation of the TST reaction, making it unreliable [3,6]. And the diagnosis of latent TB infection solely based on TST reaction also faced similar difficulties.

Periodic chest x-ray is not a cost-effective preventive practice and is not a recommended measure. Only an initial chest x-ray is recommended for those with positive TST reaction and repeat chest x-ray only if the person developed symptoms that could be attributed to TB.

Periodic education and training on TB infection control should be carried out to ensure the understanding of all HCWs regarding their occupational risks and the appropriate infection control measures.

### Engineering control

Engineering control is another important element in TB infection control. This measure uses environmental methods to reduce the concentration of droplet nuclei in the air. Inadequate ventilation or recirculation of air has been identified as a contributory factor in nosocomial transmission of TB. As the UMMC has a central air-conditioning, it is therefore important to ensure that the air from the isolation room is not recirculated into the central system. Negative pressure with six air changes per hour is the recommended requirement for TB isolation room [6]. For areas without central air-conditioning, efforts to maximise natural ventilation through open windows should be made [4].

Other engineering control measures include air-disinfection techniques, such as installation of ultraviolet lights and HEPA filters. These measures are much more expensive and there is no published data to demonstrate their cost-effectiveness [3,4,6].

### Personal respiratory protection

Personal respiratory protection is another important measure that is recommended in the policy. N95 particulate respirators are provided for HCWs who have direct contact with TB patients. This mask has the ability to filter particles of up to 1 µm in size and with a filter efficiency of at least 95%. However, routine fit testing recommended by the American guidelines is not conducted, as the benefit is still questionable.

### Ongoing monitoring for efficacy

Ongoing monitoring to ensure the efficacy of the infection control measures is made possible by the surveillance program for TB in UMMC. This surveillance was introduced in 2001 and should enable us to evaluate the effectiveness of our control programmes.

## CONCLUSIONS

Due attention should be given to TB in the healthcare setting because of the heavy disease burden in the community as well as in hospitals. Specific guidelines on preventive measures for ambulatory care setting, including radiology clinics, should be developed to enable HCWs working in those areas to reduce the risk of infection.
